# Viscoelastic Behavior of Embroidered Scaffolds for ACL Tissue Engineering Made of PLA and P(LA-CL) After In Vitro Degradation

**DOI:** 10.3390/ijms20184655

**Published:** 2019-09-19

**Authors:** Judith Hahn, Gundula Schulze-Tanzil, Michaela Schröpfer, Michael Meyer, Clemens Gögele, Mariann Hoyer, Axel Spickenheuer, Gert Heinrich, Annette Breier

**Affiliations:** 1 Leibniz-Institut für Polymerforschung Dresden e. V., 01069 Dresden, Germany; spickenheuer@ipfdd.de (A.S.); gheinrich@ipfdd.de (G.H.); breier@ipfdd.de (A.B.); 2 Institute of Anatomy and Cell Biology, Paracelsus Medical Private University, Salzburg and Nuremberg, 90419 Nuremberg, Germany; gundula.schulze@pmu.ac.at (G.S.-T.); clemens.goegele@pmu.ac.at (C.G.); 3 Forschungsinstitut für Leder und Kunststoffbahnen (FILK) gGmbH, 09599 Freiberg, Germany; michaela.schroepfer@filkfreiberg.de (M.S.); michael.meyer@filkfreiberg.de (M.M.); 4 Department of Biosciences, Paris Lodron University Salzburg, 5020 Salzburg, Austria; 5 Amedes MVZ für Laboratoriumsdiagnostik und Mikrobiologie Halle/Leipzig GmbH, 06112 Halle, Germany; mariann.hoyer@amedes-group.com; 6 Technische Universität Dresden, Faculty of Mechanical Science and Engineering, Institute of Textile Machinery and High Performance Material Technology, 01069 Dresden, Germany

**Keywords:** anterior cruciate ligament, viscoelastic behavior, tissue engineering, PLA, P(LA-CL), embroidered scaffold, ligamentocytes, cytoskeleton

## Abstract

A rupture of the anterior cruciate ligament (ACL) is the most common knee ligament injury. Current applied reconstruction methods have limitations in terms of graft availability and mechanical properties. A new approach could be the use of a tissue engineering construct that temporarily reflects the mechanical properties of native ligament tissues and acts as a carrier structure for cell seeding. In this study, embroidered scaffolds composed of polylactic acid (PLA) and poly(lactic-co-ε-caprolactone) (P(LA-CL)) threads were tested mechanically for their viscoelastic behavior under in vitro degradation. The relaxation behavior of both scaffold types (*moco*: mono-component scaffold made of PLA threads, *bico*: bi-component scaffold made of PLA and P(LA-CL) threads) was comparable to native lapine ACL. Most of the lapine ACL cells survived 32 days of cell culture and grew along the fibers. Cell vitality was comparable for *moco* and *bico* scaffolds. Lapine ACL cells were able to adhere to the polymer surfaces and spread along the threads throughout the scaffold. The mechanical behavior of degrading matrices with and without cells showed no significant differences. These results demonstrate the potential of embroidered scaffolds as an ACL tissue engineering approach.

## 1. Introduction

The anterior cruciate ligament (ACL) is the most commonly injured anatomical structure of the knee joint (47%) [1]. Surgical reconstruction using autologous tissue from hamstring or patellar tendons (PT) is recommended due to impaired knee stability, resulting in non-physiological mobility and degenerative changes when the ACL is ruptured. Nevertheless, limitations including autograft availability and deviating biomechanics, occurring when substituting a ligament (e.g. the ACL) with tendons, are causing a steadily growing interest in the research area of tissue engineering [2,3,4]. An ideal biomaterial substitute should mimic the structure and function of the native ACL until the scaffold is replaced with novel tissue. Thus, it should consist of biocompatible materials and exhibit a similar mechanical behavior compared to viscoelastic native ACL tissue [4,5,6,7,8]. 

Collagen type I is the main component of the extracellular matrix (ECM) of ligaments. As a typical representative of dense connective tissue, it serves to transmit forces between bones. The composition and organization of the collagen with fibrils and fibers determine the specific mechanical behavior of ligaments, such as the ACL. The fibrils are highly oriented in a wavy and nearly continuous arrangement to directly transfer stresses and strains. The hierarchical structure formed by fibrils, fibers, and fascicles, as well as the intimate interaction of collagen with other ECM macromolecules are responsible for the characteristic load-elongation (absorption of high loadings with simultaneously low deformation) and viscoelastic behavior [9,10,11,12]. 

The latter is evident by a time- and load-history dependent response to applied stresses and is determined by creep, relaxation, or hysteresis. Different reports on the viscoelasticity of native ligament and tendon tissue have demonstrated a non-linear elastic behavior in addition to a viscous behavior with two typical phenomena: (i) short-term memory effects, where the stress depends on strain rate [13]; and (ii) long-term memory effects, where the stress relaxes on a longer timescale, as evidenced by relaxation tests [5,14]. 

Danto and Woo (1993) investigated the stress-strain behavior of lapine ACL and PT to compare the response to different strain rates between 0.003 mm/s and 113 mm/s [13]. The modulus of the ACL was less sensitive to strain rate changes, compared to the PT and demonstrated an increase of 31%. 

Pioletti and Rakotomanana (2000) tested human ACL and PT with four different load values on a constant elongation rate of 0.3 mm/s and demonstrated fast relaxation immediately after strain application, a moderate relaxation between 500–1000 s and slight relaxation after that [14]. 

Panjabi and Courtney (2001) developed a test protocol to investigate the influence of high-speed subfailure stress on changes in the elastic, failure, and viscoelastic properties of the lapine ACL, and demonstrated an increase in deformation in load-deformation tests and a decrease in stress in the relaxation tests [15]. 

Literature on the viscoelastic behavior of particular textile scaffolds for ligament tissue engineering is rare. Most of the published results on braided [16,17,18,19], knitted [20,21,22], or twisted [23,24] textiles originate from investigations of the mechanical parameters under static conditions. Round braided structures seem to have been considered most often in the context of investigations on ligament tissue engineering in comparison with other textile manufacturing techniques, and data on the relaxation behavior have also been published [25,26]. 

Freeman et al. (2009) demonstrated a comparable stress relaxation behavior for braid-twist scaffolds made of PLA and native ACL tissue, whereby normalized stresses differed significantly after 1000 s in a braid-twist PLA scaffold with 87%, compared to native ACL with 63% [26]. 

In another study on braid-twist scaffolds made of collagen type I fibers, Walthers et al. (2012) demonstrated that the mechanical properties of cross-linked scaffolds without gelatin were closest to human ACL compared to other scaffold groups in the study. Nevertheless, mechanical compliance and ductility needed to be strongly adjusted [25]. Relaxation tests were performed on non-degraded scaffolds in both studies. 

However, when using polymeric biomaterials for tissue engineering applications, degradation processes and their influence on mechanical properties are of great importance. 

Therefore, the aim of this study was to investigate the viscoelastic properties of embroidered scaffolds made of PLA and P(LA-CL) after in vitro degradation. Two scaffold types—*moco* (mono-component scaffold solely made of PLA threads) and *bico* (bi-component scaffold made of P(LA-CL) and PLA threads)—were selected in previous studies as optimal in terms of mechanical [27,28] and biological [29,30] properties. Some of the scaffolds were seeded and cultivated with lapine ACL cells for up to 32 days. Relaxation behavior was investigated and the conditions were comparable to a set-up used by Panjabi and Courtney [15] for lapine ACL. Hence, a comparison between artificial embroidered scaffolds and native tissue is possible based on the results obtained in this study.

## 2. Results

### 2.1. Relaxation Behavior during Hydrolytic Degradation

**Figure 1:**
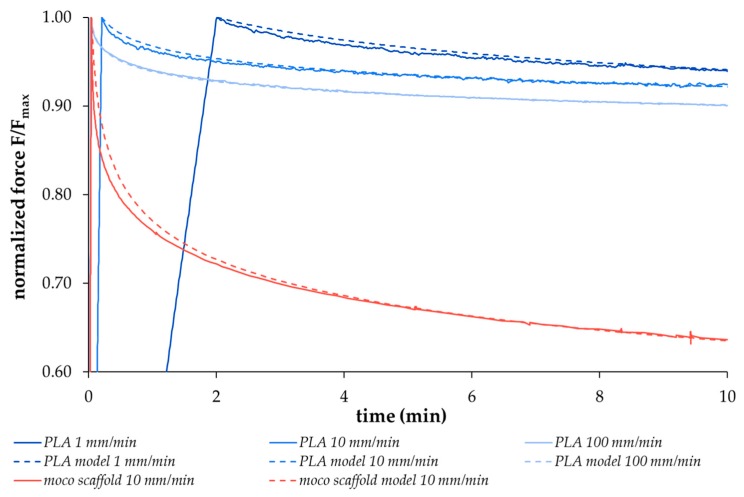
Exemplary relaxation behavior of PLA multifilament threads at different loading speeds (1 mm/min, 10 mm/min, 100 mm/min, solid blue curves) as well as of a *moco* scaffold at 10 mm/min (measured data: solid red curves) compared to the non-linear regression model curves (R^2^ > 0.98, model data: blue and red dashed curves).

**Figure 2:**
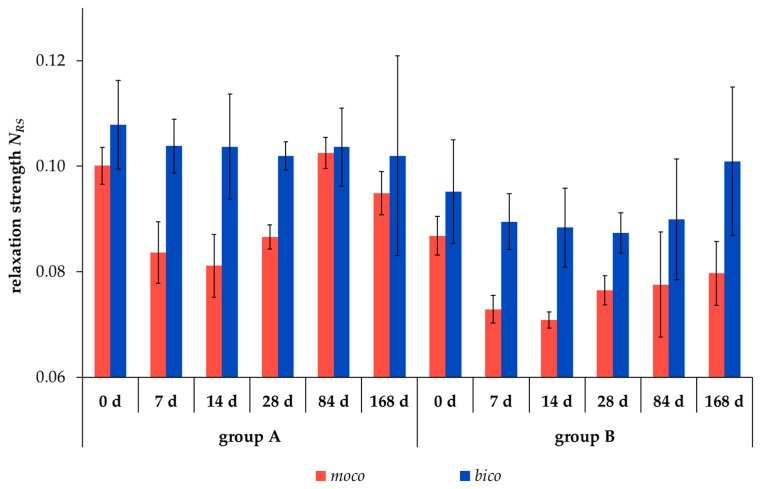
Relaxation strength *N_RS_* (dimensionless variable) of unseeded scaffold types *moco* (red) and *bico* (blue) in test groups A (without preconditioning) and B (Group B: preconditioning with 10 hysteresis cycles) during hydrolytic degradation for 0 to 168 days (d) (mean ± SD).

The relaxation behavior of different viscoelastic materials, including the degradable polymers used in this study, demonstrates force-time curves that can only be simulated with non-linear models. These models are used to ensure comparability and reproduce the respective material properties. The replication of the relaxation behavior of the two scaffold types with a high degree of agreement was realized with a non-linear regression model proposed by Obukhov (1968) [31], selected in preliminary experiments. In the first step, both thread materials were stressed at different loading speeds (1 mm/min, 10 mm/min, 100 mm/min) up to a defined elongation; the loss of force over time was then recorded. Figure 1 shows exemplary data for the PLA thread (solid blue curves) and the corresponding regression curves (dashed blue curves) as well as an example of a *moco* scaffold at 10 mm/min loading speed (red curves, solid: measured data, dashed: model). The respective curves were reproduced using the regression model (R² > 0.98). An increasing deviation of the model curves directly after reaching the initial time *t_in_* occurs with an increasing loading speed from 1 mm/min to 100 mm/min for the PLA multifilament. In the second step, the dimensionless variable *N_RS_* (0 (weak relaxation) < *N_RS_* << 1 (strong relaxation)) was determined by using the underlying formula of the regression model (see Section 4.7), which allows to predict the strength of the relaxation. This variable was then used to compare the relaxation behavior of the two scaffold types with different degradation times (0, 7, 14, 28, 84, 168 days) and pre-loading conditions (Group A without preconditioning, Group B with hysteresis preconditioning). The results are presented in Figure 2. 

The relaxation strength *N*_RS_ significantly decreased after preconditioning of the scaffolds by 10 hysteresis cycles, comparing the test groups A and B for both scaffold types at almost all degradation measuring points (*p* < 0.05, except for *bico* after 84 and 168 days). Scaffold type *moco* exhibited a lower relaxation strength compared to *bico* in both test groups and at all degradation measuring points. The lowest value of the relaxation strength was observed for *moco* and *bico* scaffolds after 14 and 28 days, respectively.

### 2.2. Comparison of the Relaxation Behavior between Scaffolds and Native Lapine ACL Tissue 

**Figure 3:**
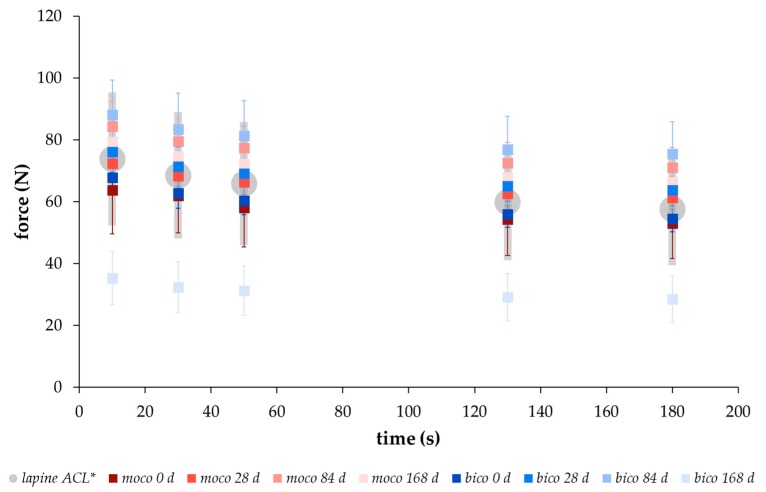
Relaxation behavior from 0 s to 180 s of unseeded *moco* (red) and *bico* (blue) scaffolds after different degradation measuring points (0, 28, 84, 168 days (d)) compared to the behavior of lapine ACL (gray) measured by * Panjabi and Courtney [15] (force-time behavior of test group B with hysteresis preconditioning, mean ± SD).

In order to compare the relaxation behavior of the embroidered scaffolds with native tissue, the force values were determined after hysteresis preconditioning and stressed with a deformation of 0.75 mm at 10 s, 30 s, 50 s, 130 s, and 180 s, according to the testing conditions of Panjabi and Courtney for native lapine ACL tissue [15]. A similar relaxation behavior with force values within the range of the standard deviation of native lapine ACL could be demonstrated for both scaffold types (Figure 3). An increase in the force values was measured from 0 to 84 days of degradation with maximum at day 84, followed by a decrease at day 168 for both scaffold types. The data in Table 1 was compiled in order to make a statement on the short- (<180 s), medium- (180 s to 300 s), and long-term (>300 s) relaxation behavior of the embroidered scaffolds. Between 10 s and 180 s of the relaxation test, the force values decreased for both scaffold types at each degradation measuring point. The highest decrease of the force value was determined after 168 days with 23% and 16% for *bico* and *moco* scaffolds, respectively. In comparison, medium- and long-term relaxation behavior was slightly lower, showing a decrease in the force for both scaffold types and at all degradation measuring points of approx. 5% and 6% for *moco* and *bico*, respectively.

### 2.3. Ultimate Tensile Properties of Degraded Scaffolds after Preconditioning

Ultimate tensile load (UTL in N), ultimate tensile elongation (UTE in %), and stiffness (S in N/mm) were determined according to Hahner et al. (2015) [27]. Values for completely non-preconditioned embroidered scaffolds after hydrolytic long-term degradation can be taken from Hahn et al. (2017) [32] for comparison. A possible influence of certain preloading conditions on the characteristic maximum values of the scaffolds shall be pointed out based on the presented data. The results of test groups A and B for *moco* and *bico* scaffolds at different degradation measuring points are shown in Table 2. With the exception of the stiffness values of *bico* scaffolds after 168 days of hydrolytic degradation (significance *p* < 0.05), no significant differences of UTL, UTE, or S values between Group A and Group B could be found. Both scaffold types exhibited a significant decrease in UTL and UTE values after 168 days, whereas the stiffness values increased significantly. 

### 2.4. Lapine ACL Cell Survival on Embroidered Scaffolds

**Figure 4:**
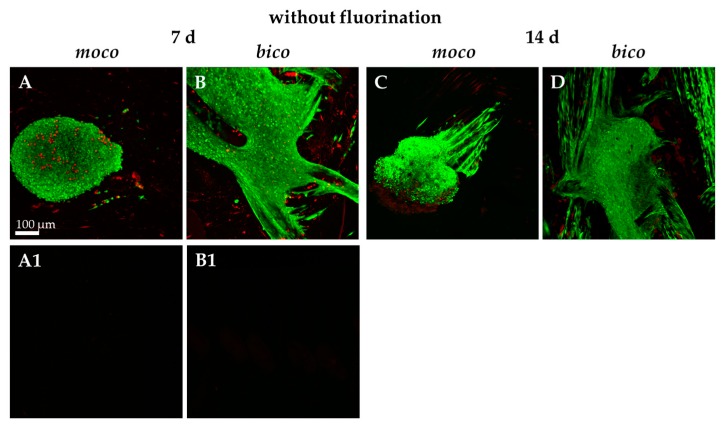
Confocal laser scanning microscopy (CLSM) of vitality staining of *moco* and *bico* scaffolds without fluorination seeded with spheroids of lapine ACL cells for 7 (**A**,**B**) and 14 (**C**,**D**) days (d). Vital cells are stained green, dead cells are shown in red. (**A1**,**B1**) The background autofluorescence of unseeded scaffolds is shown. Scale bar: 100 µm.

Due to the favorable results of previous investigations on mechanical properties, cell-seeded scaffolds were examined and characterized. To determine the adherence and spreading of lapine ACL cells on the embroidered scaffolds, sterilized embroidered scaffolds were seeded with self-assembled spheroids of lapine ACL cells (Figure 4A–D). 

**Figure 5:**
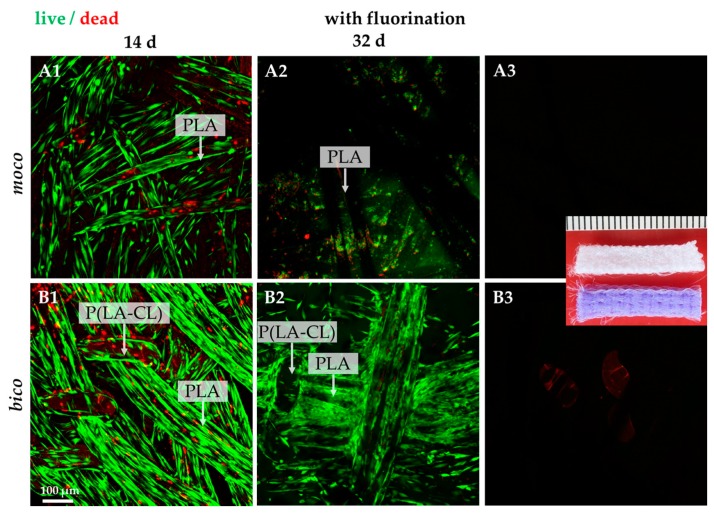
Vitality staining of fluorinated *moco* and *bico* scaffolds seeded with lapine ACL cells for 14 and 32 days (d). Vital cells are stained green, dead cells are shown in red. (**A3**,**B3**) The background autofluorescence of unseeded fluorinated scaffolds is shown. Scale bar: 100 µm (**A1**–**B3**). Inset: macroscopical image of unseeded scaffolds used in this study, scale bar: 1 mm.

It was observed that pure PLA scaffolds (*moco*) allowed only poor spheroid adherence and no cell spreading after one week of cultivation. The highest degree of cell adherence and scaffold colonization by migrating cells was detected on pure PCL scaffolds; however, the combination of PLA with P(LA-CL) (*bico*) increased the degree of spheroid-mediated scaffold colonization. Therefore, the scaffolds were functionalized by gaseous fluorine. Most of the cells survived on both scaffolds over the whole observation period of 32 days (Figure 5(A1–B3)). Only a very small number of dead cells could be identified on the threads. Cells aligned in the direction of the threads. No difference in cell vitality was observed when comparing *moco* and *bico* scaffolds after 14 days. Several cells on the *moco* scaffolds were later (day 32) found to be detached, in contrast to the *bico* scaffolds.

### 2.5. Protein Expression of Lapine ACL Cells on the Scaffolds in Comparison to Native ACLs

**Figure 6:**
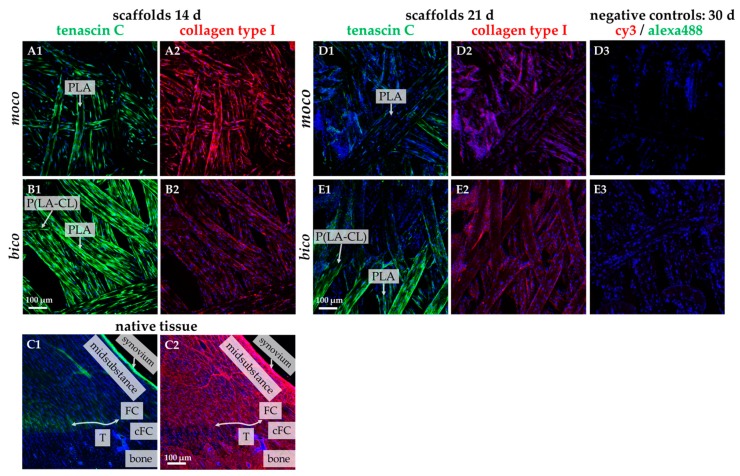
Tenascin C and collagen type I immunolabeling of lapine ACL cells seeded on fluorinated *moco* and *bico* scaffolds for 14 days and 21 days (d), compared to native ACL tissue with enthesis. Tenascin C: green. Collagen type I: red. (**C1**,**C2**) native lapine ACL tissue. cFC: calcified fibrocartilage, FC: fibrocartilage, T: tidemark (indicated by an double-headed arrow). (**D3**,**E3**) negative staining controls. Cell nuclei: blue. Scale bars (**A1**–**E3**) 100 µm.

The fibroblast marker tenascin C and type I collagen were labeled on the scaffolds seeded with lapine ACL cells as the most abundant ECM component in ligaments (Figure 6). Comparing both scaffold types after 14 days, *moco* scaffolds appeared to contain more type I collagen, whereas the *bico* scaffolds exhibited more tenascin C (Figure 6(A1–B2)). Both ECM proteins could also be demonstrated after 21 days with more pronounced expression in *bico* scaffolds (Figure 6(D1–E2)). The collagen type I expression was more pronounced in the midsubstance of the native rabbit ACL compared to both scaffold types. The synovial lining revealed an intensive collagen type I reactivity, whereas the fibrocartilage zone of the enthesis was only weakly immunolabeled (Figure 6(C1,C2)). 

**Figure 7:**
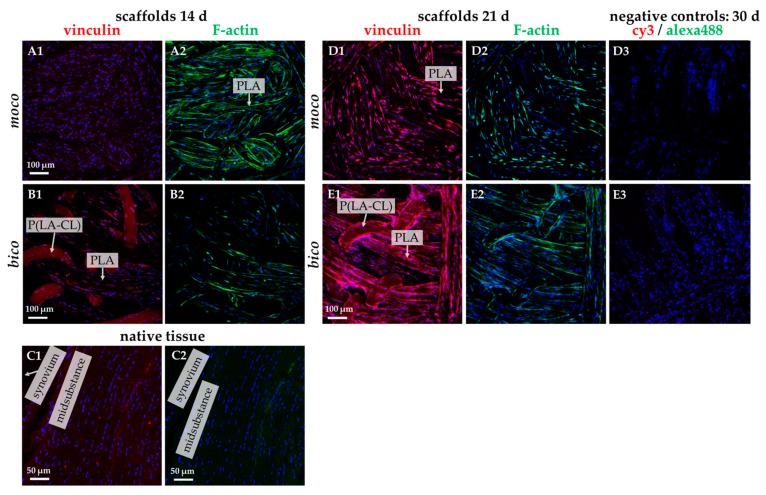
Vinculin and F-actin immunolabeling of lapine ACL cells seeded on fluorinated *moco* and *bico* scaffolds for 14 days and 21 days (d), compared to native ACL tissue. (**C1**,**C2**) native lapine ACL tissue. Vinculin: red. F-actin: green. Cell nuclei: blue. (**D3**,**E3**) negative staining controls. Scale bars (**A1**–**E3**) 100 µm, scale bars (**C1**,**C2**) 50 µm.

The F-actin/vinculin staining revealed the orientation of the cells in the direction of the scaffold threads. The cells formed cytoskeletal F-actin fiber bundles during the observation (Figure 7(A1–E3)). Vinculin immunolabeling became more evident on day 21 (Figure 7(D1–E2)). Interestingly, vinculin and F-actin could barely be seen in the native ACL (Figure 7(C1,C2)).

### 2.6. Mechanical Properties of Cell-Seeded Scaffolds

**Figure 8:**
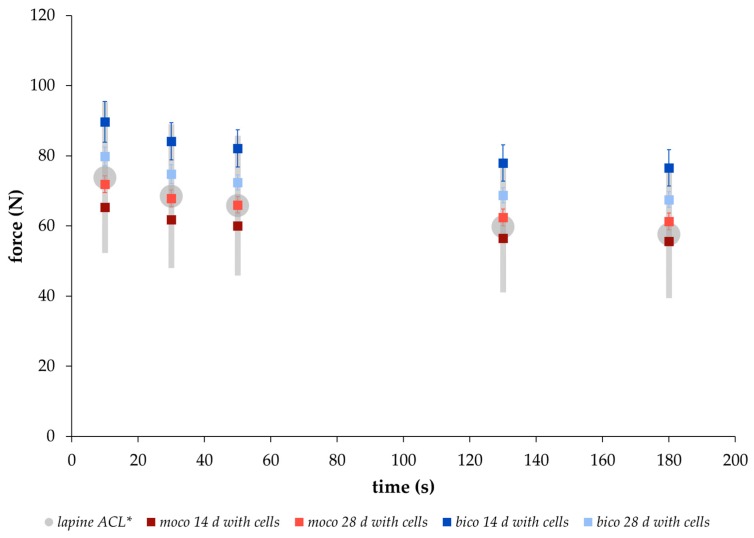
Relaxation behavior from 0 s to 180 s of cell-seeded *moco* (red) and *bico* (blue) scaffolds after 14 and 28 days (d) in vitro cultivation, compared to the behavior of lapine ACL (gray) measured by * Panjabi and Courtney [15] (mean ± SD).

Based on the results of the preconditioning experiment (Section 2.3) with unseeded scaffolds showing no significant differences for ultimate tensile values between test groups A and B, the viscoelastic properties of scaffolds seeded with lapine ACL cells were determined for test group B only. Therewith, a comparison to the relaxation behavior of native lapine ACL tissue (data from Panjabi and Courtney (2001) [15]) was also possible. After 14 and 28 days under in vitro degradation, the relaxation behavior of both scaffold types was within the range of the native lapine ACL values [15] (Figure 8).

**Figure 9:**
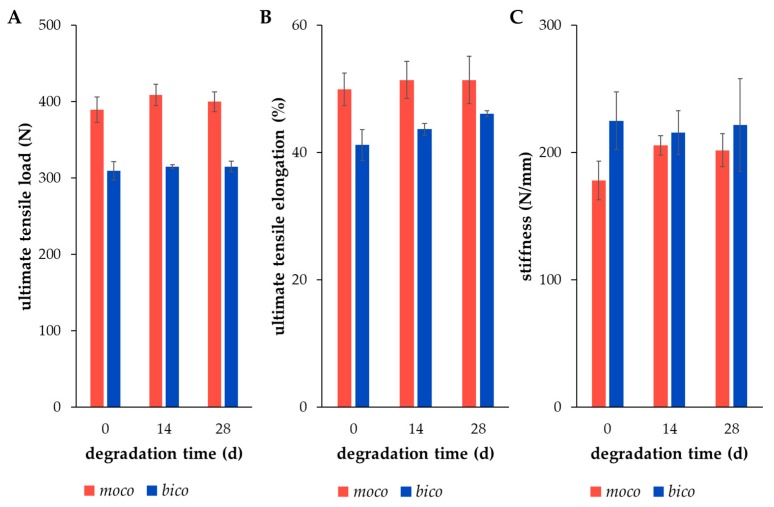
Ultimate tensile properties ((**A**) UTL in N, (**B**) UTE in %, (**C**) S in N/mm) of embroidered, fluorinated, and ethylene-oxide (EO) gas sterilized *moco* (red) and *bico* (blue) scaffolds in dry state (day 0), and in vitro seeded with lapine ACL cells for 14 and 28 days (d) (mean ± SD).

Furthermore, the results of Section 2.4. and Section 2.5. show a relevant number of cells on the scaffold, deducing the assumption that the degradation process might be affected by the cells; however, the ultimate tensile properties provide no clear evidence of this, as compared to the scaffolds that are degraded only hydrolytically. Therefore, the ultimate tensile properties of cell-seeded embroidered *moco* and *bico* scaffolds were investigated without any preconditioning by the relaxation test or hysteresis. There were no significant differences in the mechanical values when comparing dry state and degraded state after 14 and 28 days, respectively (Figure 9). Additionally, the values of UTL, UTE and S were comparable to values of unseeded scaffolds after similar degradation periods under hydrolytic conditions [32].

Thus, even under cell culture conditions, the embroidered scaffolds demonstrate promising mechanical behavior, comparable to that of native ACL tissue.

## 3. Discussion

Tissue-engineered scaffolds for ACL replacement should be biocompatible and biodegradable, enable adequate cell growth, and provide a mechanical behavior comparable to native tissue. Previous studies have already established strategies for cell seeding [30,33], as well as adjustment of the mechanical and structural properties [27,28] of the embroidered structures, and have demonstrated the potential of this application in the field of ligament tissue engineering.

The aim of this study was to investigate the alteration of the viscoelastic behavior of embroidered scaffolds made of polymeric thread materials PLA and P(LA-CL) during in vitro degradation in comparison to the mechanical properties of native lapine ACL tissue.

Embroidery technology was chosen for scaffold preparation due to its high flexibility and freedom of design [27], facilitating the manufacturing of two different types of scaffolds. These scaffolds differ in their material composition in the upper and lower threads (*moco* consisting of PLA in the upper and lower thread; *bico* consisting of P(LA-CL) in the upper and PLA in the lower thread). In both these scaffolds, mechanical tests were carried out to determine viscoelastic properties, in addition to cell colonization experiments. All the embroidered scaffolds used in this study showed dimensions comparable to native lapine ACL tissue [34].

However, further focus of this study was on mechanical investigations of the scaffolds’ viscoelastic behavior. Viscoelasticity is a key feature required for function. Therefore, the relaxation behavior of both scaffold types, *moco* and *bico,* was measured and compared to a regression model proposed by Obukhov (1968) [31] for quantitative comparison. First, to check the applicability of the model, the respective thread materials and the embroidered scaffolds were stressed with different loading speeds up to an elongation of 5%, which is within the physiological range of elongation of the ACL [35]. Threads and embroidered scaffolds showed typical strain rate dependent stress behavior. The applicability was confirmed with a good correlation between the regression model and the mechanical data (coefficient of determination R² > 0.99 (Figure 1)). In addition, a comparison of the relaxation strength at different degradation points for both scaffold types was made using the regression model (R² > 0.93). The decrease in relaxation strength of the preconditioned structures (Group B vs. Group A) is explained by the alignment of the thread materials in the embroidered structure in the direction of force induced by the cyclic loading applied during hysteresis. The *moco* scaffolds tended to have a lower relaxation strength, compared to the *bico* scaffolds, due to the use of P(LA-CL) as an additional thread material in the latter. P(LA-CL) has a different chemical and molecular structure, molecular weight, and architecture, which influences the viscoelastic behavior of polymers [36]. The relaxation behavior of both scaffold types exhibited fast (0–180 s) and moderate (180–600 s) relaxation immediately after 5% elongation was applied to the scaffolds. This is similar to the results of a study by Pioletti and Rakotomanana (2000) on native human ACL tissue [14]. If the cross-sectional area of their investigated structures (native tissue vs. textiles) is taken into account, human ACL with 44.4 mm² and a force of about 300 N results in stress of 7 MPa [14]. The braid-twist scaffold made of PLLA examined by Freeman et al. (2009), with a cross-sectional area of 0.8455 mm² and 8 N, corresponds to about 9 MPa [26]. The *moco* and *bico* scaffolds in this study showed an average cross-sectional area of about 8 mm² and force values between 70–90 N, resulting in an estimated stress of 8–11 MPa. Thus, the values of embroidered scaffolds are similar to other textile structures (such as braids) as well as native ACL tissue. In addition, the relaxation tests of test group B were carried out in a manner comparable to the protocol of Panjabi and Courtney (2001) on native lapine ACL [15]. An equivalent relaxation behavior was observed for unseeded (Figure 3) and cell-seeded (Figure 9) embroidered scaffolds.

Initial cell seeding using spheroids revealed only poor cell adherence on non-functionalized *moco* compared to *bico* scaffolds. On *bico*, more cells migrated from the spheroids on the scaffold and colonized the threads, suggesting that the P(LA-CL) provides more suitable binding opportunities for the cells than pure PLA. Using functionalization with fluorine and a suspension culture, homogenous cell growth on both *moco* and *bico* scaffolds was achieved, suggesting that this functionalization strategy should be used in future. In the cell-seeding experiments, the *bico* scaffolds allowed the long-term survival of lapine ACL cells, whereas the pure PLA scaffolds (*moco*) did not sufficiently support long-term culture, irrespective of fluorination. Hence, in future, additional functionalization strategies, e.g., using collagen preparations, would be of advantage because of their remarkable biocompatibility [37]. As shown after three weeks on fluorinated scaffolds, the cells revealed a distinct cytoskeletal architecture with cytoskeletal F-actin filaments and focal adhesion formation, indicated by vinculin immunolabeling, which underlines an intimate cell-biomaterial interaction. Moreover, most of the cells reflected an orientation along the threads. This may be due to “contact guidance”, which leads to preferred cell growth, depending on the chemical, geometric, or mechanical conditions of the substrate [38]. However, within the native tissue, actin and vinculin could barely be detected, underlining that stress fiber formation is a feature of cell spreading in the scaffold. ACL cells in scaffold culture also produced typical ECM components such as type I collagen and glycoprotein tenascin C, which remained mostly cell-associated for the duration of the observation time. In contrast to the scaffold culture, collagen type I deposition was much more pronounced in the midsubstance and non-calcified fibrocartilage but weak in the calcified fibrocartilage zone of the native ACL; however, collagen type I and tenascin C were clearly associated with the ECM. Tenascin C was only weakly expressed in situ in the ECM of the ACL midsubstance, but more intensely labeled in the tidemark region of the enthesis and especially, in the covering synovium of the ACL. These differences between scaffold culture and native tissue might become neglectable with longer observation periods of colonized scaffolds needed to achieve a more mature ECM organization.

The ultimate tensile properties—UTL, UTE and S—were also investigated to understand the influence of preconditioning with relaxation and hysteresis treatment, and the presence of cells affecting the degradation processes, respectively. There were no significant differences in the mechanical values between the test group A without and test group B with preconditioning. The long-term hydrolytic degradation behavior of the embroidered structures made of PLA and P(LA-CL) was analyzed with regards to their ultimate tensile properties, molecular weights, as well as surface structure changes demonstrated by SEM images; the results were previously published [32]. In comparison, the mechanical testing was performed without any preconditioning. Nonetheless, no significant differences of the ultimate tensile properties were identified. As long as the pre-treatment remains within the elastic deformation range of the scaffolds, there seems to be no influence on the ultimate tensile properties. Nevertheless, the increase in stiffness values during degradation, especially for the *moco* scaffolds, was also mentioned [32]. Besides, no significant changes in UTL, UTE, and S values appeared for the in vitro degraded scaffolds, compared to the non-degraded and the hydrolytically degraded ones over 28 days, irrespective of different treatment by fluorination. The influence of fluorination on thread surface structures (publication in preparation [39]), as well as on the mechanical properties [40], has been clarified in ongoing studies.

Furthermore, the values of both hydrolytically and in vitro degraded scaffolds were comparable to the values of native lapine ACL tissue with UTL of (364 ± 85) N, UTE of (32 ± 18)%, and S of (175 ± 40) N/mm [15,41,42]. Therewith, the viscoelastic behavior and degradation rate of the embroidered scaffolds were found to be in a suitable and desired range for an ideal scaffold needed for ACL tissue regeneration [43,44]. With regards to the scaling of the scaffolds to other animal or human ACL dimensions, a great advantage of embroidery technology for scaffold production is its high degree of design flexibility. The first animal experiment on nude mice and rabbit models are being planned in order to provide a statement regarding the perspective application of the embroidered structures for reconstruction of the human ACL.

## 4. Materials and Methods

### 4.1. Scaffold Fabrication

Two thread materials, a monofilament suture thread made of P(LA-CL) (USP 7-0, Gunze Ltd., Osaka, Japan) and a melt spun multifilament consisting of six filaments made of PLA (Tt = 155 dtex, pellets from NatureWorks LLC (Minnetonka, MN, USA, melt spun at the IPF Dresden, Germany) were processed using embroidery technology (JCZ 0209-550, ZSK Stickmaschinen GmbH, Krefeld, Germany) [27,45]. The scaffolds were fabricated on a water-soluble non-woven made of polyvinyl alcohol (PVA, Freudenberg Einlagestoffe KG, Weinheim, Germany). The fabric was washed in distilled water three times for 30 min each, using a compact shaker (KS 15 A, Edmund Bühler GmbH, Bodelshausen, Germany). The scaffolds (length 25 mm, wide 4 mm, thickness 2 mm; three plies locked together; pattern with stitch length 1.8 mm, stitch angle 15°, duplication shift 0.2 mm) were then dried at room temperature (RT). Two types of scaffolds—PLA/PLA as *moco* scaffold type and P(LA-CL)/PLA as *bico* scaffold type—were used in this study.

*Scaffolds for mechanical testing after hydrolytic degradation:* The procedure for long-term hydrolytic degradation (37 °C, 5% CO_2_, complete cell culture medium) was previously described in detail [32]. The scaffolds were sterilized with ethanol absolute and UV-light before placing them in six-well plates with 4 mL complete cell culture medium per well. For analysis of the mechanical properties, 10 specimens (*n* = 10, five for each mechanical test group) of each scaffold type were sampled and washed in distilled water after 7, 14, 28, 84 and 168 days.

*Scaffolds for cell culture experiments and mechanical testing after* in vitro *degradation:* The embroidered scaffolds were first fluorinated. This was realized at the FILK (Freiberg, Germany) in a fluorination batch reactor (Fluor-Technik-System GmbH, Lauterbach, Germany) with a mixture of 10% fluorine gas in air for 60 s. The samples were sterilized by ethylene-oxide (EO) gas sterilization (University Hospital Carl Gustav Carus Dresden, Dresden, Germany) before testing in cell culture. Finally, the samples (*n* = 6, three each for test group B and testing without preconditioning) were collected after 14 and 28 days from the cell culture for the mechanical testing.

### 4.2. Lapine Anterior Cruciate Ligament Cell Isolation by Explant Culture

Lapine ACL samples were explanted from dead New Zealand White (NZW) rabbits (female donors, age around 11 weeks), sacrificed in other approved experimental animal projects (including LaGeSo Berlin 396/09, 21 January 2010). Three native ACLs were fixed in 4% paraformaldehyde (PFA) and embedded in paraffin for immunohistology. For cell isolation, surrounding connective tissue of the ACLs, including the synovial membranes, was removed. The pure ligament tissue was cut into 1–2 mm^2^ slices and incubated in culture flasks (Sarstedt, Nümbrecht, Germany) with growth medium (Ham’s F-12/Dulbecco’s Modified Eagle’s (DMEM) Medium 1:1) containing 10% fetal calf serum (FCS), 10,000 IU/mL penicillin / 10,000 µg/mL streptomycin, 2.5 µg/mL amphotericin B, non-essential amino acids (all from Biochrom AG, Berlin, Germany), and 25 µg/mL ascorbic acid (Sigma-Aldrich, Munich, Germany) at 37 °C and 5% CO_2_. After 1−2 weeks, the ACL fibroblasts started to migrate from the tissue slices. Subsequently, fibroblasts were harvested using 0.05% trypsin/0.02% Ethylenediaminetetraacetic acid (EDTA) (Biochrom AG, Berlin, Germany) and sub-cultured.

### 4.3. Scaffold Seeding: Dynamical Culture

10^6^ lapine ACL cells were suspended in 5 mL growth medium (4 × 10³/mm³ per scaffold) and transferred into a Tubespin^®^ Bioreactor 50 tube (TPP^®^, Trasadingen, Switzerland) before being dynamically cultivated for 7 to 32 days on a rotatory device (Sunlab, Sustainable Lab Instruments, Aschaffenburg, Germany). Growth medium was changed 2–3 times a week. Experiments were performed in independent triplicates.

### 4.4. Scaffold Seeding: Spheroid Culture

Using the hanging drop method, spheroids consisting of 50,000 cells per spheroid were harvested after 72 h and five spheroids seeded on the scaffolds, which were pre-incubated for 30 min in a growth medium in an uncoated petri dish (nerbe plus GmbH, Winsen/Luhe, Germany). Spheroid-scaffold cultures were maintained for 7 and 14 days. Growth medium was changed 2–3 times a week.

### 4.5. Live-Death Assay

Cell vitality of ligamentocytes cultured on scaffold was visualized using a live/dead assay based on fluorescein diacetate (FDA, Sigma-Aldrich, Munich, Germany) and propidium iodide (PI, Carl Roth GmbH, Karlsruhe, Germany). After day 7, 14, and 32, the samples were incubated for 30 s in FDA/PI staining solution (15 µg/mL FDA and 10 µg/mL PI dissolved in phosphate buffered saline (PBS)). The green (living cells, fluorescein positive) and red (dead cells, PI) fluorescence was monitored using a SPE-II confocal laser scanning microscope (CLSM, Leica, Wetzlar, Germany).

### 4.6. Immunofluorescence Analysis of Marker Expression

The protein expression profile was determined using CLSM. Samples were fixed in 4% PFA and washed with Tris buffered saline (TBS: 0.05 M Tris, 0.015 M NaCl, pH 7.6), before incubation with protease-free donkey serum (5% diluted in TBS with 0.1% Triton X100 for cell permeabilization) for 20 min at RT. Paraffin sections were deparaffinized and demasked with 0.1% pronase solution. Subsequently, sections or scaffold segments were incubated with primary antibodies (mouse anti-human-tenascin C (GeneTex Inc. Biozol, Eching, Germany), goat anti-type I collagen (goat anti human, Abcam, Cambridge, UK), and mouse anti-vinculin (mouse-anti-human, Sigma-Aldrich, Munich, Germany)) overnight at 4 °C in a humidified chamber. Samples were rinsed with TBS before incubation with donkey-anti-goat or anti-rabbit-Alexa-488 (0.4 µg in TBS) or donkey-anti-mouse or goat-Cyanine3 (Cy3, both Invitrogen, Carlsbad, CA, USA) coupled secondary antibodies (diluted 0.2 µg in TBS with 0.1% Triton X100 and 5% donkey serum), respectively, for 1 h at RT in a humidified chamber. Cell nuclei were counterstained using 4′,6′-diamidino-2-phenylindol (20 µg, DAPI, Roche, Mannheim, Germany) and Phalloidin-Alexa488 (1 µg in TBS, Sigma-Aldrich, Munich, Germany) to depict F-actin cytoskeletal actin architecture. Labeled cells were washed several times with TBS, before mounting with fluoromount mounting medium (Southern Biotech, Biozol Diagnostica, Eching, Germany) and examined using a SPE-II CLSM (Leica, Wetzlar, Germany).

### 4.7. Mechanical Testing

The mechanical properties were studied by using a tensile testing machine (Z 2.5 with 1 kN load sensor, 10 mm gauge length *l_gauge_* and pneumatic metal test clamps) controlled with TestXpert software (both Zwick/Roell GmbH & Co. KG, Ulm, Germany). To investigate the applicability of the regression model, PLA and P(LA-CL) threads as well as embroidered scaffolds were stressed with different loading speeds (1 mm/min, 10 mm/min and 100 mm/min) up to an elongation of 5% and then the force drop over time was measured (*n* = 3). The non-linear regression model of Obukhov (1968) was chosen to determine the strength of the relaxation represented by the material parameter *N_RS_* to compare different scaffold materials and compositions [31]. This model is described by the following equation:

(1) $$F\left(t\right)=F_{in}\times {\left(\frac{t_{in}}{t}\right)}^{N_{RS}}$$

where *F_in_* is the maximum force at the time *t_in_* the loading was removed and *N_RS_* is a dimensionless material parameter (0 < *N_RS_* << 1).

The procedure of the mechanical testing was set up by two test groups (A and B) performing two test rounds (1 and 2) each for the hydrolytically degraded scaffolds (Table 3). Group A starts with a relaxation test (Test 1), which was performed at 0.75 mm (corresponds to an elongation of 7%) for 10 min. After a pause of 45 min, the scaffolds were loaded with 10 hysteresis cycles at 0.5 mm and then stressed to failure with 10 mm/s testing speed (test 2). Group B was preconditioned with ten hysteresis cycles at 0.5 mm before the relaxation test. Test 2 was identical in Group B as in Group A in all aspects. All loadings of the relaxation test or hysteresis cycles were performed with 10 mm/min testing speed. The scaffolds remained clamped in the tensile testing machine until failure. The test procedure for Group B (force values measured after 10 s, 30 s, 50 s, 130 s and 180 s) is comparable to the testing procedure A1 of lapine ACL from Panjabi and Courtney (2001), and was also performed with the in vitro degraded scaffolds [12].

Additionally, the cell colonized scaffolds were tested for their ultimate tensile properties without any preconditioning by relaxation or hysteresis after 14 and 28 days. For this purpose, the scaffolds were also loaded to complete failure with a test speed of 10 mm/s and the load-displacement behavior was recorded.

The ultimate tensile load *UTL* (in N), ultimate tensile elongation *UTE* (in %) (*UTE = l_max_/l_gauge_* × 100 with associated displacement *l_max_*), and stiffness *S* (in N/mm) were calculated according to [27]. The results for the mechanical properties were represented as mean ± standard deviation (mean ± SD) and significance level was set at 5%.

## 5. Conclusions

Two different scaffold types (*moco* and *bico*) were investigated in an effort to study their viscoelastic behavior in comparison with native lapine ACL tissue. Moreover, the determination of mechanical properties after degradation of unseeded and ACL cell-seeded scaffolds was performed. Cell adhesion and growth could be improved by functionalizing the surfaces of the scaffolds with fluorine. In conclusion, the outcomes of the mechanical tests performed in this study underline the fact that the embroidered structures are mechanically stable and resistant. The investigations with cell-colonized scaffolds suggest that a similar slow degradation could be expected in further in vivo experiments.

## Figures and Tables

**Table 1:** Force values (F) measured at different times (10 s, 180 s, 300 s and 600 s) and degradation measuring points (0, 7, 14, 28, 84 and 168 days (d)) for both scaffold types, *moco* and *bico,* in test group B (mean ± SD).

	Relaxation Time (s)	Force (N) at Different Degradation Measuring Points (Days) (Mean ± SD)					
		0	7	14	28	84	168
*moco*	10	63.8 ± 14.2	67.8 ± 6.6	66.6 ± 5.4	72.4 ± 7.7	84.4 ± 7.9	79.3 ± 6.6
	180	53.0 ± 11.5	58.0 ± 5.4	56.8 ± 4.7	61.4 ± 6.9	71.1 ± 6.4	66.5 ± 5.8
	300	51.2 ± 11.1	56.2 ± 5.2	55.2 ± 4.5	59.6 ± 6.6	68.8 ± 6.1	64.8 ± 5.4
	600	48.6 ± 10.7	53.9 ± 5.0	53.0 ± 4.4	57.1 ± 6.4	65.8 ± 5.9	61.7 ± 5.6
*bico*	10	67.8 ± 5.0	71.6 ± 5.8	73.6 ± 7.6	76.2 ± 6.8	88.1 ± 11.3	35.4 ± 8.7
	180	54.5 ± 4.2	59.4 ± 5.0	61.1 ± 7.1	63.7 ± 6.2	75.5 ± 10.4	28.5 ± 7.5
	300	52.2 ± 4.0	57.5 ± 4.9	59.3 ± 6.9	61.8 ± 5.9	73.5 ± 10.1	27.4 ± 7.3
	600	49.2 ± 4.0	54.8 ± 4.6	56.7 ± 6.7	59.2 ± 5.8	70.7 ± 9.8	26.1 ± 7.1

**Table 2:** Influence of hysteresis preconditioning on the ultimate tensile properties (UTL in N, UTE in %, S in N/mm) of *moco* and *bico* scaffolds after hydrolytic degradation (0, 7, 14, 28, 84 and 168 days (d)) (mean ± SD).

		*moco*			*bico*		
		UTL (N)	UTE (%)	S (N/mm)	UTL (N)	UTE (%)	S (N/mm)
Group A	0 d	405.8 ± 12.1	53.6 ± 5.4	151.0 ± 23.0	334.4 ± 14.8	40.2 ± 3.0	196.0 ± 19.2
	7 d	394.6 ± 16.0	62.4 ± 2.6	170.8 ± 22.4	338.1 ± 6.0	55.1 ± 1.1	255.4 ± 18.6
	14 d	409.3 ± 17.9	62.2 ± 3.5	202.2 ± 29.8	323.4 ± 17.5	53.5 ± 2.6	239.1 ± 31.5
	28 d	388.1 ± 13.4	58.6 ± 5.6	210.7 ± 36.0	326.5 ± 12.9	54.9 ± 2.4	281.2 ± 9.2
	84 d	360.4 ± 5.0	41.8 ± 1.8	224.8 ± 14.3	290.6 ± 15.0	43.4 ± 2.3	206.9 ± 34.5
	168 d	330.0 ± 9.6	44.0 ± 2.7	261.8 ± 27.9	232.6 ± 36.5	46.8 ± 5.5	259.8 ± 70.5
Group B	0 d	401.2 ± 19.8	50.4 ± 7.2	160.2 ± 28.8	333.1 ± 7.7	39.4 ± 2.6	192.0 ± 8.6
	7 d	410.1 ± 15.3	61.6 ± 3.2	204.3 ± 17.1	329.4 ± 19.0	53.0 ± 3.9	240.7 ± 9.0
	14 d	402.2 ± 13.6	61.2 ± 3.2	199.8 ± 19.6	319.2 ± 9.4	52.4 ± 2.3	246.8 ± 14.2
	28 d	394.9 ± 8.7	58.4 ± 3.0	218.3 ± 21.9	323.0 ± 16.5	53.0 ± 2.6	254.7 ± 14.6
	84 d	348.7 ± 6.8	43.8 ± 2.0	192.6 ± 11.8	295.9 ± 15.6	45.6 ± 2.1	231.3 ± 46.4
	168 d	329.2 ± 29.8	44.8 ± 4.5	235.4 ± 26.7	210.9 ± 27.8	47.0 ± 5.8	114.7 ± 31.9

**Table 3:** Mechanical testing procedure of embroidered scaffolds after hydrolytic degradation. Both test groups A and B were performed in two sequences with a pause of 45 min between test 1 and 2. During the pause, the scaffolds remained in a tensile testing machine. In group A, the relaxation test was performed at 0.75 mm (initial loading speed 10 mm/min) for 10 min, followed by a hysteresis with 10 cycles (cyc, 0.5 mm with speed of 10 mm/min) and a single stretch to failure with 10 mm/s. Group B differs from group A with 10 hysteresis cycles (0.5 mm with speed of 10 mm/min), which serve as preconditioning before the relaxation test.

Test	Group A	Group B ^1^
1	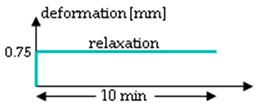	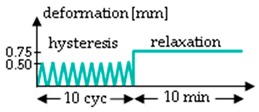
pause	45 min	
2	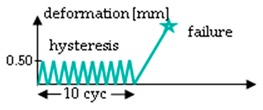	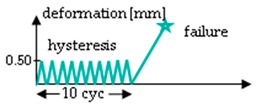

^1^ Testing conditions of Group B are comparable to testing procedure A1 from Panjabi and Courtney (2001), which was performed on lapine ACL [15].

## Abbreviations

2D	Two-dimensional
3D	Three-dimensional
ACL	Anterior cruciate ligament
*bico*	Bi-component scaffold made of PLA as upper thread and P(LA-CL) as lower thread
DAPI	4′,6-diamidino-2-phenylindole
DMEM	Dulbecco’s Modified Eagle’s
ECM	Extracellular matrix
EO	Ethylene-oxide gas sterilization
FCS	Fetal calf serum
FDA	Fluorescein diacetate
*moco*	Mono-component scaffold made of PLA threads
NZW	New Zealand White rabbit
PBS	Phosphate buffered saline
PFA	Paraformaldehyde
PI	Propidium iodide
PLA	Polylactic acid
P(LA-CL)	Poly(lactic-co-ε-caprolactone)
PT	Patellar tendon
RT	Room temperature
S	Stiffness in N/mm
SD	Standard deviation
TBS	TRIS buffered saline
TE	Tissue engineering
UTE	Ultimate tensile elongation in %
UTL	Ultimate tensile load in N
